# The Causes Which Affect the Sanitary Condition of Chicago

**Published:** 1859-06

**Authors:** DeLaskie Miller

**Affiliations:** President of the Chicago Academy of Medical Sciences


THE CHICAGO

MEDICAL JOURNAL.

VOL. II. JUNE, 1859. No. 6.

### ORIGINAL COMMUNICATIONS.

ARTICLE I.

### THE CAUSES WHICH AFFECT THE SANITARY CONDITION OF CHICAGO.

By DELASKIE MILLER, M. D., President of the Chicago Academy of Medical Sciences.

AN INAUGURAL ADDRESS, ON ASSUMING THE CHAIR.

GENTLEMEN:—At this, the first regular meeting of the Chicago Academy of Medical Sciences, some remarks upon the objects and benefits of our organization might be considered appropriate. But the objects aimed at in organizing this Society must, I am sure, be fully comprehended, at least by the members. We seek *practical* results. To reach these, the Academy has been divided into different sections, appropriate to the investigation of the different departments of medical science. One of these, "On Public Health, etc.," to which I, with others, have been assigned, has suggested to me, as a suitable subject for this evening, *The Causes which Affect the Sanitary Condition of Chicago.*

This subject, as I think, comes legitimately before us for consideration.

The preservation of health, and the attainment of longevity, are objects of desire to all. And the physician can act most efficiently in promoting these objects, when the causes are fully understood, which would destroy the one or abbreviate the other.

Although much has been written upon the sanitary condition of this city, still, I have not, as yet, seen all the circumstances stated, which bear directly upon the subject, or their influence properly estimated. Partial views must ever lead to erroneous conclusions. And thus we can readily understand why such a diversity of opinion prevails in the public mind, especially abroad, upon the sanitary condition of Chicago.

I might here refer to the official reports of our city mortality for a series of years, and thus easily settle the question of its insalubrity or healthfulness, relatively, as compared with other cities ; but this is not now my object.

My business now is, to enumerate the circumstances, and describe the mode in which these circumstances affect the health of this city. If there are causes which exist within our limits, or around us, which render this locality insalubrious, it is important that they be made public, that their evil consequences may be obviated.

The topography of the city, then, in the first place, claims our attention. The site of this city is such as to impress the minds of those unacquainted with the surrounding circumstances, most unfavorably. It is, to appearance, a level plain, elevated only a few feet above the surface of Lake Michigan, which bounds it on the east. The soil is porous, composed of a light sand and alluvium, which rests on an impervious clay bed.

This character of the soil favors the rapid absorption of the water which is deposited upon its surface, but which is held near the surface by its inability to penetrate the bed which immediately underlies. And although the water may rapidly disappear from sight, it is nevertheless held ever ready to be elevated into the atmosphere again, by any circumstance which favors evaporation, bringing up with it the malaria and deleterious gases generated by animal and vegetable decomposition, which is continually taking place in a city large as this.

An atmosphere highly charged with aqueous vapors, is the efficient vehicle for transporting miasmata. Here is a potent cause of disease, which would produce its injurious effects in various ways. 1st. The atmosphere, which, during the warm season, would be charged with aqueous vapors nearly to saturation, would, by constantly acting upon the system, produce derangement and debility, by interfering with the chemical changes which should take place in the blood by respiration, and which is manifested by indisposition to engage in either physical or mental labor. Also, in this condition, the system is less able to resist the general causes of disease. 2d. To this must be added the effects produced by the poisonous materials, introduced directly into the blood through the lungs in respiration, which derange the blood, and, through it, produce disease in the solids of the body.

I have thus spoken of the causes connected with the *natural site* of the city, which tend to generate disease and would favor its extension. They are such as impress themselves most readily upon the mind, and, doubtless, have had a great influence in coloring the opinion so frequently expressed, of the insalubrity of this city, if, indeed, they have not been the only views taken of the subject. But, when we extend our observation further, we shall find that there are counteracting circumstances, which greatly modify the views taken above, and go far to neutralize the effects of these general causes of disease. I will enumerate some of them. But, first, I must be allowed to say, that, were the city immediately surrounded by lofty forests, or high mountain ranges, which would prevent the free movement of the atmosphere, and which would allow the air, charged with the poisonous exhalations, to rest on the city, and to be inhaled by its inhabitants, the consequences would not vary from the effects enumerated above.

But, fortunately for the city, this is not the case. Its location is most favorable, having on the east and north-east Lake Michigan, including an area of about 13,500 square miles of nearly chemically pure water, permitting no unhealthy exhalations from its surface, and allowing the freest movements of the purest winds over the city, and through the straight, broad streets, which come daily, charged with elements to invigorate the body, and to displace the unwholesome atmosphere generated within our limits. Or, do the winds rush in from the opposite direction, they also come to us equally pure, having traversed a level or slightly undulating prairie, which extends beyond us to an almost unlimited extent, unbroken by pools of stagnant water, or miasmatic marshes, but "with verdure clad."

From these causes, instead of being condemned to breathe the pestilential vapors, which are always generated in a large city, and are fruitful sources of disease, we here, from our natural position, may inhale the pure breezes of nature without stint. This is a condition most essential to health and longevity; for, without it, all other advantages are valueless.

Thus has nature herself removed from us, more effectually than man could do, the disadvantages which might, at first sight, appear insuperable.

This great natural advantage of our location as a city, cannot be too highly estimated. If we inquire into the causes which render any locality insalubrious, we will always find pre-eminent among them, a deficient supply of pure air, or circumstances preventing the free movements of the atmosphere, so as to displace rapidly the impure, by air of a better quality.

We live on air, and would we live well, this should be pure in quality, and unlimited in quantity.

Next in importance to pure air, is an abundant supply of good water. Of the great value of water, in a hygienic point of view, I surely need not stop here to speak. The supply of water has, for centuries, been the great desideratum in large towns, which, many times, it has been impossible to obtain. In many places, the inhabitants are compelled to depend upon the water afforded by wells. Water obtained in this way, in large cities, can never be wholesome, no matter how much the natural circumstances of location, or geological formations, may favor it. These wells usually occupy the lots on which the residences and outhouses are erected, and the quality of the water, of necessity, must be greatly influenced by the gases from sewers, privies, cesspools, etc., which find their way through the soil to it. Add to this the lime, and various saline and mineral substances frequently met with, and we have a combination of causes not calculated to render the water innocuous. But here, again, Chicago owes much to her natural position, on the shore of Lake Michigan, which affords us an abundant supply of water, and of an excellent quality; notwithstanding, at times, by the action of the waves, it is delivered to us holding the minutest particles of clay in suspension, rendering it milky in appearance, and unpleasant to the eye. But this does not render it as obnoxious, either to the smell or taste, as has ofttimes been represented. I apprehend that the greatest objection to it, is, in reality, its appearance, contrasted with its usual crystalline transparency; for, I am not aware, that the amount of clay which it contains, at the most unfavorable periods, is so very deleterious to the system, as is by many imagined; besides, these periods are usually of short duration, and the foreign matter can, even then, with the greatest facility, be perfectly separated, by filtering.

Another circumstance worthy of notice is, that the water for the city is taken from the lake nearly a mile north of the mouth of the river.

On the western shore of the lake, a current constantly flows from north to south, as is evidenced by deposits on the north side of piers, when extended into the lake. This, of course, prevents the impure water of the river from being taken up and distributed for the use of the city.

The water taken from the lake is now distributed over a large part of the city; so that here the poor even, may enjoy the luxury of a plentiful supply of excellent water—denied to the wealthy in many places.

I wish now to direct your attention to another question, rather suggestively than as giving any elaborated views of my own. It is this: What effect has Chicago River upon the health of the city? It passes, as you know, through the main part of the city, for about three-fourths of a mile, then branches extend to the extreme southern and north-western limits, having usually but little current, and receiving the decomposing filth that always collects in considerable quantities along its shores; besides, the sewers are so constructed as to lead into it from the different sections of the city. In this way a large amount of material, calculated to produce unfavorable results, is constantly carried into its waters.

Does the river, under these circumstances, produce an unfavorable effect upon the public health? As I before stated, I have not the data to enable me to give a decided opinion. It would not, however, be difficult to determine the question accurately; for, we frequently have strong winds, which prevail for several days successively, in the same direction, and would enable us to note the effects upon the different sides of the river. This, repeated a number of times in different seasons of the year, would enable us to arrive at satisfactory conclusions. The subject, I think, is worthy the attention of the members of the profession, and, if it should be found to be manifestly productive of disease in its immediate vicinity, then, suggestions regarding the remedy could come from no more proper source than this Academy.

In the early part of this paper, while speaking of the site of the city, I enumerated the circumstances which, in themselves, strongly predispose to disease, especially such diseases as have been denominated zymotic. Now, removal beyond the circle of their influence, is sure to give immunity from an attack; and, also, the removal of the cause, or causes, is equally certain to give immunity from the disease. Can this be effected in Chicago? Can we overcome the disadvantages of a low surface and porous soil? I answer, Yes. The present high grade, recently established, which raises the streets from two to eight feet above the natural level, is a most efficient means; then, the character of much of the material used in grading, preparatory to paving, which is a clay and gravel, so mixed as to make a bed well nigh impervious to water, preventing absorption, and which also arrests the ascent of noxious exhalations. Then add to this the benefits of the system of sewerage, also but recently commenced, but which is being carried forward most actively: and we comprise all the conditions necessary to obviate the objections to the natural site of the city.

I cannot refrain here, from referring to the improper construction of the dwellings, especially of the poorer classes, and the excessive crowding of numerous persons into small and illy ventilated apartments, so frequently met with here, and producing here as elsewhere, its baneful effects. Every physician, and none but the physician, who has frequent opportunities to witness this evil, can fully appreciate the disastrous consequences of this total neglect of the simplest principles of ventilation and want of room. How frequently do we find numbers of persons crowded into a single apartment, which rests directly on the damp surface beneath, without the slightest provision in its construction for ventilation, which serves them for parlor, kitchen and bedroom, outraging every principle of common decency, to say nothing of the laws of health; and in addition, in the cold season especially, every crevice, which might admit a small amount of pure air, is carefully closed; thus completely excluding this, the most essential element of man's existence, except what is accidentally admitted by the door, which is opened for as short a period as possible, to afford ingress or egress to the inmates.

In vain for them has nature kindly supplied an abundance of this vitalizing element for every creature — in vain the gentle breezes, freighted with materials to invigorate the body—to lend brilliancy to the eye—to the cheek the ruddy glow of health—and give to the brain activity and energy, to incite to "deeds of noble daring." They prize not the blessing, but close the door against it.

I have collected a few statistics of the mortality of this city, for several years past, by which we are enabled to judge of the relative mortality as compared with several other cities. By these, we will be enabled to judge whether the surrounding circumstances, favoring a free supply of pure air, an abundance of water, etc., when joined to the benefits to be derived from the grading of the city, paving and complete sewerage, are not to remove the odium of the insalubrity, so long and persistently attached to this city. I give the results only. They are based upon the official census, as far as I could obtain them, and the record of interments, as kept by the City Sexton. They read as follows:

| | | | | | | |
|---|---|---|---|---|---|---|
| In the year | 1846, | there was 1 death | in every | | 43.33 | persons. |
| | 1849, | 1 | " | " | 18.84 | " |
| | 1850, | 1 | " | " | 21.45 | " |

In '49 and '50, the city was visited by the cholera in its severest form, as may be seen by comparing those years with 1846.

But the record of mortality here, for the past four years, gives us the most favorable results :

| | | | | | | |
|---|---|---|---|---|---|---|
| In 1855 | there was | 1 | death for | every | 42.4 | persons. |
| 1856 | " | 1 | " | " | 38 | " |
| 1857 | " | 1 | " | " | 55.3 | " |
| 1858 | " | 1 | " | " | 56 | " |

If you will compare these figures with similar figures of the most favored cities of the country, you will find that Chicago suffers no disparagement by the comparison ; and this is Chicago, too, before she has had the benefits of the improvements to which I have alluded — for these have, as yet, been but just commenced.

I have taken the liberty of copying from the 9th vol. of the Transactions of American Medical Association, the following :

In Philadelphia, the proportion of deaths was as follows—

| | | | |
|---|---|---|---|
| In 1852, | 1 | in every | 40.10 |
| 1853, | 1 | " | 43.61 |
| 1854, | 1 | " | 38.10 |
| 1845, | 1 | " | 47.81 |

In the city of Boston, for

| | | | |
|---|---|---|---|
| 1850, | 1 | in every | 37.84 |
| 1855, | 1 | " | 39.88 |

In New Orleans, the average from 1846 to 1854 inclusive, 1 in every 15.7.

In Baltimore, the average of deaths was, from 1836 to 1848 inclusive, 1 in every 42.72.

I will here invite your attention to the impression which so generally prevails in the public mind, in regard to the infant mortality of this city. Should we take our cue from editorials in the city papers, or from the remarks so frequently made by many citizens, we should be led to infer that it was next to impossible for children to survive in Chicago. Doubtless, the infant mortality of Chicago, as in all other large cities, is too great, which seems to be, and truly is, enormous. But does this city suffer, in this respect, by comparison with other cities ? It may not be generally known, that, of all the deaths which occur in cities, one-half are among children under five years of age.

But, by referring to the statistics of Philadelphia, New York, Boston, London, and some of the principal cities on the continent, this is found to be the case. I have examined the official reports of mortality for this city, for the years 1855, 1856, 1857, and 1858, and find that the mortality here, among the children under five years of age, is about equal to that of the city of Philadelphia, which is conceded to be one of the most healthful cities in this country. Then, again, it appears to me fair, to refer to another circumstance, which would have an influence upon these figures, in our favor. Our community is composed mostly of young persons, and a larger proportion of them are raising families than in other cities. Then, of course, we should have, relatively, a larger proportion of children, to augment the infant mortality here.

The subject of infant mortality introduces us directly to the consideration of its causes. Of all these, I cannot, of course, speak, in the time to which propriety would limit me. But, one potent cause demands our attention here. *The adulterated milk furnished to cities*, and a large part of it for the sustenance of infants. The infant organization is such, that the slightest cause is liable to destroy its powers of assimilation, and its hold upon life so feeble, that it rapidly sinks, when imperfectly nourished.

Now, the milk, daily furnished to cities, is not only largely diluted, but so greatly adulterated, as to render it unfit for use. In saying this, I am aware that I am but repeating what has been said before. Still, the evil prevails, to an alarming extent; and, it is for this reason, I allude to it now.

We have legal enactments, restraining the sale of unwholesome food, and especially of adulterated milk. Why do not the proper authorities enforce its provisions upon this traffic? This, surely, is of the greatest importance, affecting the happiness of thousands, and striking at the tenderest ties in society; for, its greatest physical evils are visited upon infancy.

This subject may seem trite to the members of this Academy; but, if the above are facts, their destructive consequences are not a whit lessened, because the subject is trite. And, if public excitements are powerless in remedying the evil, because, like spasmodic efforts, they suddenly relax; or, if legal enactments are ineffectual, because the public do not realize the enormity of the evil; then, surely, on the members of our profession must rest, to a very great extent, the responsibility; for to them the public prosperity looks, as the conservators of the public health. By our united and personal efforts, in the same direction, we may effect much towards mitigating the evil, if, indeed, we do not entirely remove it.

Gentlemen: I have thus enumerated, in as brief a manner as I could, the causes which affect the sanitary condition of this city. I have not been biased in these remarks, by favor or prejudice, to give undue weight to one, or under-estimate the influence of another class of causes. I have had no theory which would lead my judgment astray, or interest to color my opinions. I have endeavored to follow where facts and unquestioned principles would conduct. To what conclusion, then, have we arrived?—to this: That the location of Chicago, so favorable for commercial and physical prosperity, is equally favorable for the preservation of health and attainment of longevity. That, when the present system of municipal improvements, now being carried forward with commendable energy, should be completed, we may rationally expect that this city will occupy the position, in regard to its sanitary advantages, that it now does in its physical prosperity, and be acknowledged without a parallel.

